# Differential Alterations in the Expression of Neurotransmitter Receptors in Inner Retina following Loss of Photoreceptors in rd1 Mouse

**DOI:** 10.1371/journal.pone.0123896

**Published:** 2015-04-02

**Authors:** Prerna Srivastava, Sumit K. Sinha-Mahapatra, Abhinaba Ghosh, Ipsit Srivastava, Narender K. Dhingra

**Affiliations:** National Brain Research Centre, Manesar (Gurgaon) Haryana, India; National Eye Institute, UNITED STATES

## Abstract

Loss of photoreceptors leads to significant remodeling in inner retina of rd1 mouse, a widely used model of retinal degeneration. Several morphological and physiological alterations occur in the second- and third-order retinal neurons. Synaptic activity in the excitatory bipolar cells and the predominantly inhibitory amacrine cells is enhanced. Retinal ganglion cells (RGCs) exhibit hyperactivity and aberrant spiking pattern, which adversely affects the quality of signals they can carry to the brain. To further understand the pathophysiology of retinal degeneration, and how it may lead to aberrant spiking in RGCs, we asked how loss of photoreceptors affects some of the neurotransmitter receptors in rd1 mouse. Using Western blotting, we measured the levels of several neurotransmitter receptors in adult rd1 mouse retina. We found significantly higher levels of AMPA, glycine and GABAa receptors, but lower levels of GABAc receptors in rd1 mouse than in wild-type. Since GABAa receptor is expressed in several retinal layers, we employed quantitative immunohistochemistry to measure GABAa receptor levels in specific retinal layers. We found that the levels of GABAa receptors in inner plexiform layer of wild-type and rd1 mice were similar, whereas those in outer plexiform layer and inner nuclear layer combined were higher in rd1 mouse. Specifically, we found that the number of GABAa-immunoreactive somas in the inner nuclear layer of rd1 mouse retina was significantly higher than in wild-type. These findings provide further insights into neurochemical remodeling in the inner retina of rd1 mouse, and how it might lead to oscillatory activity in RGCs.

## Introduction

Loss of photoreceptors results in significant morphological and physiological alterations in the inner retina of patients and animal models of retinal degeneration [1–6]. In rd1 mouse, a widely used model of retinal degeneration, rods start to degenerate at around postnatal day 10, whereas cone degeneration starts later and continues over several months [7–8]. By adulthood, nearly all rods and a majority of cones are lost, and typically a single layer of cells remains in the outer nuclear layer [7–8]. As a result, many retinal ganglion cells (RGCs) exhibit spontaneous oscillatory bursts of spikes, which compromise the ability of RGCs to transmit visual information to the brain [9–11]. Even though the exact mechanism underlying the oscillatory activity in RGCs is not completely clear, it has been shown to be presynaptic in origin [10–15]. Levels of synaptic proteins and synaptic activity in the presynaptic bipolar cells and amacrine cells are increased following photoreceptor loss [12–13,16–17]. This is consistent with the increased levels of glutamate and GABA observed after photoreceptor loss in rd1 mouse retina [18–19].

The increased synaptic activity in the excitatory bipolar cells and the predominantly inhibitory amacrine cells, which are connected in a feedback loop, could produce oscillatory activity in the inner retina of rd1 mouse [17]. However, it is not clear how the increased synaptic activity in these cells matches with the changes in the corresponding postsynaptic neurotransmitter receptors. Furthermore, presence of multiple excitatory and inhibitory receptor types with differential cellular expression in retina makes it difficult to predict the nature of specific changes in their levels. Here, we asked how loss of photoreceptors affects the levels of some of the neurotransmitter receptors in adult rd1 mouse. Using Western blotting and quantitative immunohistochemistry, we measured the expression levels of AMPA, glycine, GABAa and GABAc receptors in adult rd1 and wild-type mice. These receptors, which are expressed widely in mouse retina and have been studied well for their expression and function, were selected to broadly represent excitatory (glutamate) and inhibitory (glycine, GABA) neurotransmitters. Glutamate, glycine and GABA receptors are the major neurotransmitter receptors present in retina [20]. The results would expand our knowledge about neurochemical remodeling in inner retina in adult rd1 mouse, and help understand the mechanisms underlying the oscillatory activity in RGCs following photoreceptor loss.

The neurotransmitter receptors AMPA, glycine, GABAa and GABAc are ligand-gated channels. AMPA receptor comprises subunits GluR1 to GluR4 which form a tetrameric structure [21]. Glycine receptor (GlyR) is composed of α (four isoforms: α1, α2, α3, α4) and β subunits (single isoform) which together form a pentameric complex in various combinations [22]. GABAaR comprises several subunits which form heteromeric complexes, but in retina the predominant subunits are α1, β2/3 and γ2 [23]. GABAc receptor (GABAcR) comprises three subunits: ρ1, ρ2 and ρ3, which form heteromeric or homomeric complexes of mainly ρ1 and ρ2 subunits in retina [24]. In human retina, the expression levels of mRNA of ρ2 subunit are two-fold higher than of ρ1 [24].

## Materials and Methods

### Ethics statement

All experiments were approved by the Institutional Animal Ethics Committee of the National Brain Research Centre. All efforts were made to minimize the number of animals used and their suffering.

### Animals and tissue preparation

Wild-type (C57BL/6J) and rd1 (PDE6b^rd1^; CBA/J) mice were obtained from Jackson Laboratory (Bar Harbor, USA), and bred locally at the animal facility of the National Brain Research Centre, India. Animals were maintained on a 12-hour light:dark cycle. Only adult animals (2–3 months old) were used in this study.

For cryosectioning, an eyeball was removed after cervical dislocation, given a small incision, and hemisected to prepare an eyecup. The eyecup was fixed (10 min for GlyR and GluR1 immunohistochemistry or one hour for GABAaR) in 4% paraformaldehyde (PFA) at 4°C. The eyecup was then immersed in 30% sucrose in phosphate-buffered saline (PBS) for one hour at 4°C for cryopreservation. The eyecup was embedded in Optimal Cutting Temperature compound and vertical sections of 10 μm thickness were cut using a cryostat (model CM3050S, Leica, Wetzlar, Germany).

For protein extraction, an eyeball was hemisected in ice-cold PBS containing 10 mM ethylenediaminetetraacetic acid (EDTA; pH 8.0) and retinas were collected in protein lysis buffer (75 μl per retina) containing 50 mM Tris (pH 7.5), 150 mM sodium chloride, 1 mM EDTA, 50 mM sodium fluoride, 1 mM sodium orthovandate, 2% sodium dodecyl sulfate (SDS) and a cocktail of protease inhibitors (Complete Protease Inhibitor cocktail; Roche Applied Science, Penzberg, Germany) on ice. Retinas were then sonicated and centrifuged at 12000 rpm for 30 minutes at 4°C. Supernatant was collected and protein levels were estimated using bicinchoninic acid method (BCA kit, Sigma-Aldrich, St. Louis, USA).

### Primary antibody characterization

**Fig 1:**
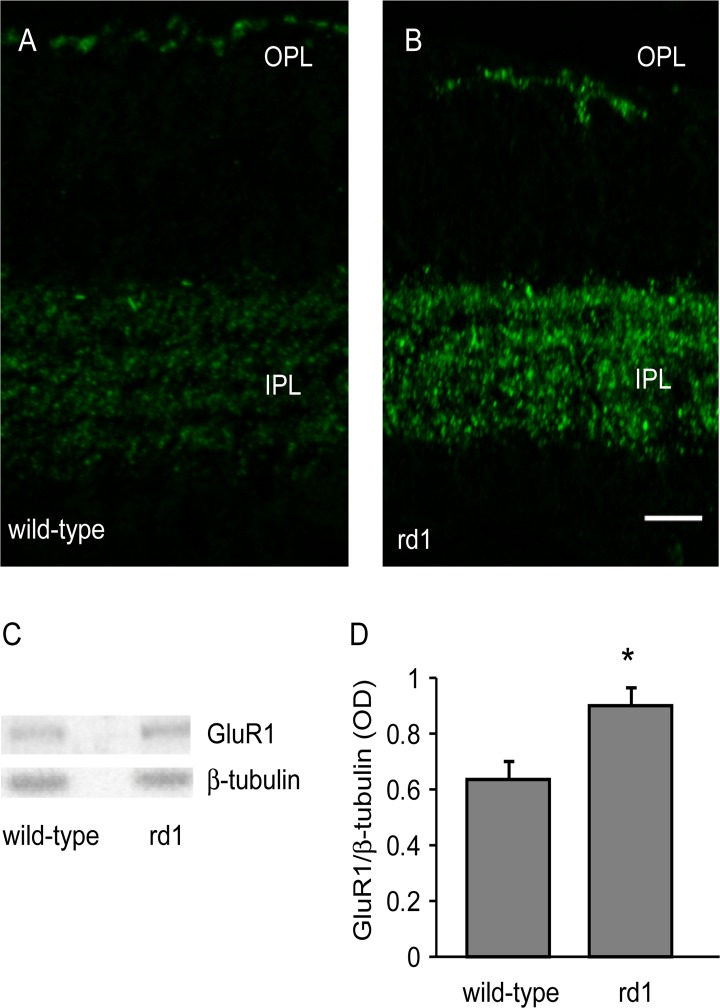
GluR1 was upregulated in rd1 mouse retina. A, B) Representative images of retinal sections of adult wild-type (A) and rd1 mouse (B) retinas immunostained for GluR1. Scale bar: 20 μm. C) Representative blots of GluR1 and β-tubulin in wild-type and rd1 mouse. D) Levels of GluR1 (mean±SD) was significantly higher in rd1 mouse retina than in wild-type (n = 10). For this and all other figures, ‘n’ represents number of animals. * p<0.05.

**Fig 2:**
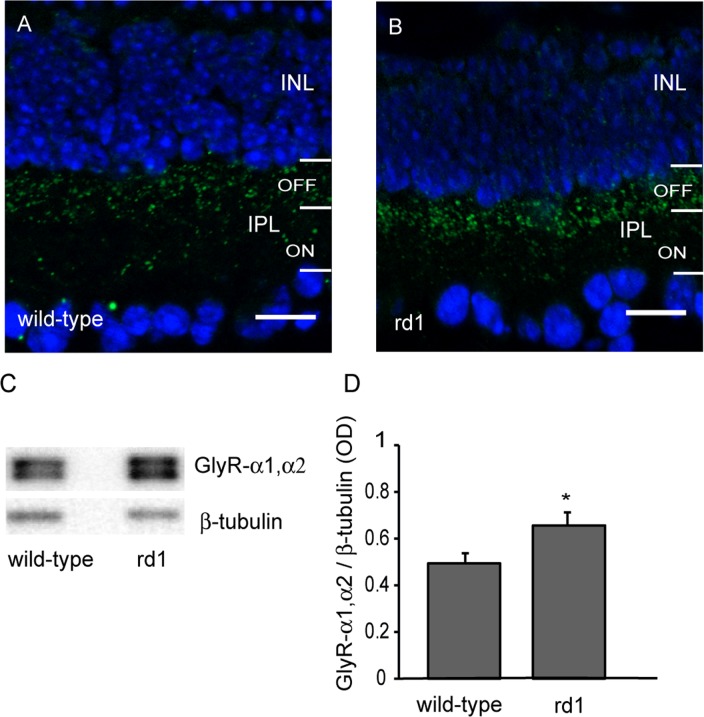
Glycine receptor was upregulated after loss of photoreceptors. A, B) Representative images of retinal sections of adult wild-type (A) and rd1 mouse (B) retinas immunostained for GlyR-α1,α2. Scale bar: 20 μm. C) Representative blots of GlyR-α1,α2 and β-tubulin in wild-type and rd1 mouse retinas. D) The levels of GlyR-α1,α2 (mean±SD) was significantly higher in rd1 mouse retina than in wild-type (n = 10) * p<0.05.

The primary antibodies used here are shown in Table 1. The AMPA receptor antibody was a rabbit polyclonal that recognizes glutamate receptor 1 (GluR1), and detects a single band at 106 kDa (manufacturer’s data; see Fig 1C). Immunolabeling showed punctate staining in outer plexiform layer (OPL) and inner plexiform layer (IPL) of mouse retina [25] (see Fig 1A and 1B). GlyR antibody was rabbit polyclonal which recognizes both α1 and α2 subunits of the receptor and detects a band near 48 kDa (manufacturer’s data; see Fig 2C). In some samples, we observed two close bands near 48 kDa, possibly representing the two subunits (not illustrated). A strong punctate GlyR labeling in the OFF sublamina of the IPL further confirmed its specificity [25] (see Fig 2A and 2B).

**Table 1:** List of Antibodies.

Antibody	Immunogen	Manufacturer, Catalog #	Species, type	dilution
GluR1	Synthetic linear peptide	Millipore (Bilerica, USA) Cat# AB1504	Rabbit polyclonal	1:4000 (IB) 1:5000 (IHC)
GlyR (α1 and α2 subunit)	Synthetic peptide of N-terminal of rat	Abcam (Cambridge, UK) Cat# AB23809	Rabbit polyclonal	1:40,000 (IB) 1:2000 (IHC)
GABAaR (α1 subunit)	A highly purified peptide QPSQDELKDNTTVFTR(C) corresponding to 28–43 amino acids of rat GABAa receptor α1 subunit with an additional C-terminal cysteine.	Sigma-Aldrich (St. Louis, USA) Cat# G4416	Rabbit polyclonal	1:2500 (IB) 1:1000 (IHC)
GABAcR (ρ2 subunit)	A peptide mapping within a cytoplasmic domain of GABAc ρ2 of mouse.	Santa Cruz Biotechnology (Santa Cruz, CA) Cat# SC-21343	Goat polyclonal	1:1000 (IB)
β-tubulin	Tubulin from rat brain	Sigma-Aldrich Cat# T4026	Mouse Monoclonal	1:15000 (IB)

**Fig 3:**
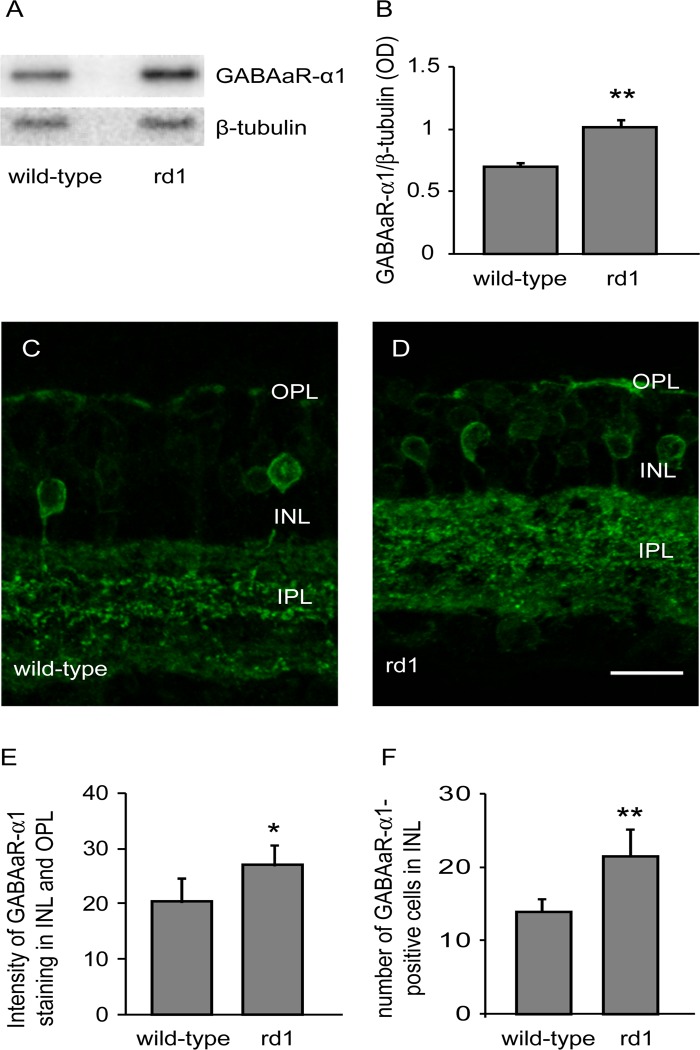
GABAaR was upregulated in rd1 mouse retina. A) Representative blots of GABAaR-α1 and β-tubulin in adult wild-type and rd1 mouse retinas. B) The expression levels of GABAa (GABAaR-α1 to β-tubulin ratio; mean±SD) were significantly higher in rd1 mouse retina than in wild-type (n = 10). C, D) Representative images of retinal sections of wild-type (C) and rd1 (D) mouse retinas immunostained for GABAaR-α1. Many somas in INL were also labeled. Scale bar: 20 μm. E) Levels of GABAaR-α1 (mean±SD) in INL and OPL were higher of rd1 mouse retina than in wild-type (n = 5). F) Number of somas expressing GABAaR-α1 in INL (mean±SD) of wild-type and rd1 mice. The number of these cells in rd1 mouse retina was significantly higher than in wild-type (n = 5). * p<0.05; ** p<0.005.

GABAaR antibody was rabbit polyclonal that recognizes α1 subunit of the receptor, and detects a single band at approximately 50 kDa in brain and retina [26–27] (manufacturer’s data; see Fig 3A). In wild-type mouse retina, it produces punctate labeling in IPL, but also labels OPL and some bipolar cell and amacrine cell somas in INL [25,28] (See Fig 3C).

**Fig 4:**
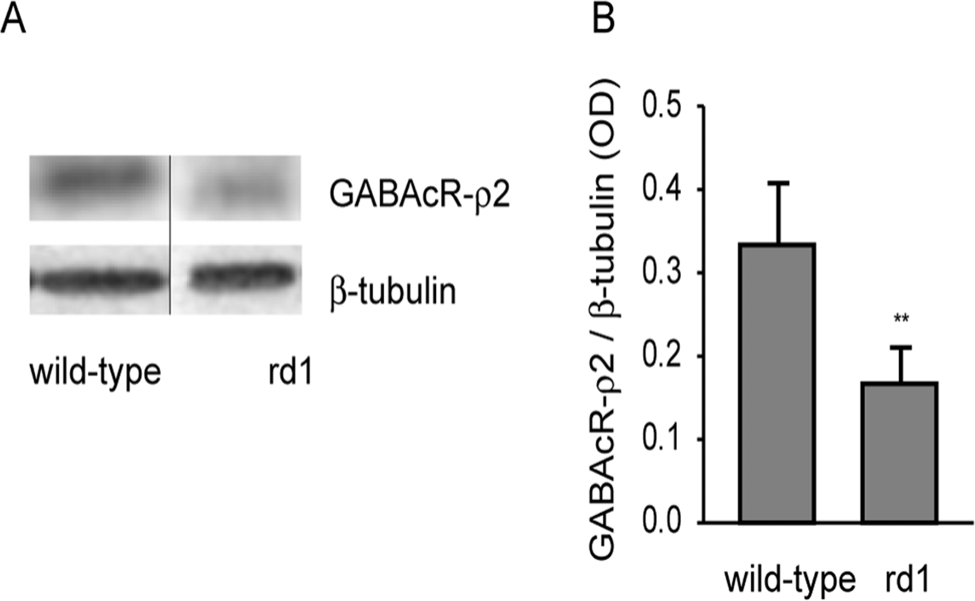
GABAcR-ρ2 levels were downregulated in rd1 mouse retina. A) Representative blots of GABAcR-ρ2 and β-tubulin in adult wild-type and rd1 mouse retinas. Black vertical line indicates that the two bands were not adjacent. B) Levels of GABAcR-ρ2 (mean±SD) were significantly lower in rd1 mouse retina than in wild-type (n = 8). ** p<0.005.

The goat polyclonal GABAcR antibody recognizes ρ2 subunit of the receptor and detects a single band at 50 kDa in mouse brain (manufacturer’s data). In mouse retina, this antibody produced a single band near 57 kDa in our hands, probably representing a specific GABAc isoform (manufacture’s data/ emails; http://www.uniprot.org/uniprot/P47742; see Fig 4A). This band disappeared when we preincubated the antibody with the relevant peptide antigen (sc-21343P; Santa Cruz). β-Tubulin antibody was used as loading control for Western blotting. This antibody detects a single band at 55 kDa which was used for normalization when quantifying the proteins.

### Western blotting

For each set of samples, equal volume containing 10 μg or 20 μg of protein sample was loaded and the proteins were separated by SDS-PAGE and subsequently transferred to an activated PVDF membrane (MDI Membrane Technologies, Ambala, India). The blots were first incubated in a blocking solution (5% BSA in Tris-buffered saline containing 0.1% Tween-20 (TBST); pH 7.4) followed by overnight incubation in a primary antibody prepared in the blocking solution, at 4°C. The blots were then washed for 3×10-min in TBST and probed with anti-rabbit or anti-goat secondary antibody conjugated with horse-radish peroxidase (Vector Laboratories) for one hour at room temperature, followed again by 3×10-min washing in TBST. The signals were detected using enhanced chemiluminescence (ECL; Millipore, Billerica, USA) with GelDoc (Universal Hood II, Bio-Rad, Hercules, USA). Densitometric analysis was performed with ImageJ software (National Institutes of Health, USA). The grayscale image was ‘‘inverted” and the background was uniformly subtracted using rolling ball radius method. The signal of each protein was normalized with the loading control, and expressed as integrated optical density (OD).

### Quantitative immunohistochemistry

Quantitative immunohistochemistry was carried out as described previously [17]. Briefly, the optimal concentration of a primary antibody was determined by using a range of antibody concentrations, plotting the staining intensity as a function of the concentration, and selecting from linear part of the curve the concentration that produced approximately half-maximum intensity. The retinal sections were incubated in a blocking solution (3% or 10% normal donkey serum (NDS), 3% bovine serum albumin (BSA) and 0.3% triton-X100 in PBS; pH 7.4) for one hour at room temperature in a humidified chamber, followed by overnight incubation in a primary antibody at 4°C. After washing 5×5-min with PBS, the sections were incubated in anti-rabbit Alexa-Fluor488-conjugated secondary antibody (Invitrogen, Carlsbad, USA) for one hour at room temperature. The sections were again washed 5×5-min with PBS and mounted in Vectashield that contained DAPI (Vector Laboratories, Burlingame, USA). Each pair of control and test samples was processed simultaneously, keeping all the conditions same. Stained samples were imaged as 1 μm thick optical serial sections in z-axis, using either an epifluorescent microscope (AxioImager Z1; Carl Zeiss, Gottingen, Germany) which was equipped with Apotome grid projection system or with Argon laser on a confocal microscope (LSM 510 Meta; Carl Zeiss, Oberkochen, Germany). The confocal parameters, such as pinhole size, amplifier offset and detector gain were optimized for control sample and then tested for the experimental sample, such that they did not produce any background noise or saturation in either. The optical sections were stacked offline to produce a composite image for intensity quantification. For each animal, five composite images were captured each from five retinal sections. The intensity values from these 25 images were averaged to generate a single value for each animal for further analysis. For quantification of the staining intensity, the region of interest in an image was marked in ImageJ, and the intensity at each pixel was measured. A histogram of these intensities typically showed two peaks: one near zero, representing the black background, and the other that was the signal of interest. To remove the background, we removed the bottom 20 values (out of 256) while the remaining values representing foreground signal were compared. Cells were counted using *cell counter* plugin in ImageJ.

### Statistical analyses

All data were analyzed using Shapiro-Wilk test in Sigmaplot (Systat Software Inc., San Jose, USA) and found to be normally distributed. Statistical comparisons between control and test samples were made using unpaired two-tailed *t-test* and p values less than 0.05 were considered significant. The data are shown as mean±SD.

## Results

### Loss of photoreceptors resulted in upregulation of GluR1

GluR1 is expressed in somas and dendrites of amacrine cells and ganglion cells, and in OFF-bipolar cell dendrites in mouse retina [25,29]. In both wild-type and rd1 mouse retinas, GluR1 was expressed in OPL and in IPL (Fig 1A and 1B). In both layers, the labeling in the rd1 mouse retina appeared more intense than in wild-type mouse.

Using Western blotting, we measured the expression levels of GluR1 in wild-type and rd1 mouse retinas. We found that the GluR1 levels in rd1 mouse (OD values; mean±SD; 0.9±0.22) were significantly higher than in wild-type control (0.64±0.21; p = 0.01; n = 10; Fig 1C and 1D).

### Loss of photoreceptors resulted in upregulation of glycine receptor

GlyR-α1 is present primarily on OFF-cone bipolar cell axon terminals and OFF ganglion cell dendrites but also on amacrine and ganglion cell processes, while GlyR-α2 is localized on bipolar cell axon terminals and amacrine cell processes [30–32].

Immunolabeling for GlyR-α1,α2 produced bright, punctate labeling in the OFF sublamina of the IPL, in both wild-type and rd1 mouse retinas (Fig 2A and 2B). Using Western blot analysis, we measured the levels of GlyR-α1,α2 in wild-type and rd1 mouse retinas. We found that the levels of GlyR-α1,α2 in rd1 mouse retina (0.66±0.16) were significantly higher than in wild-type control (0.49±0.14; p = 0.03, n = 10; Fig 2C and 2D).

### GABAa receptor was upregulated following photoreceptor loss in rd1 mouse retina

The GABAaR-α1 subunit is localized on bipolar cell dendrites in OPL, in somas of bipolar cells and amacrine cells in INL, in IPL and in ganglion cell somas [23,25,28,33]. Western blot analysis showed that the levels of GABAaR-α1 in rd1 mouse retina (1.02±0.18) were significantly higher than in wild-type (0.69±0.1; p = 0.0001, n = 10; Fig 3A and 3B).

Since GABAaR-α1 is present in several retinal layers, we asked whether the upregulation was specific to a retinal layer. We performed quantitative immunohistochemistry and measured GABAaR-α1 levels in different retinal layers. We found that the levels of GABAaR-α1 in the IPL of rd1 mouse (160.21±20.68) were statistically similar to that in the wild-type (144.37±14.22; p = 0.2). However, the levels in INL and OPL combined of rd1 mouse retina (27.12±3.84) were significantly higher than in wild-type (20.45±4.24; p = 0.03; n = 5; Fig 3C, 3D and 3E). We could not compare the GABAaR-α1 levels in OPL alone, because the OPL in the rd1 mouse appeared disintegrated in many places and selecting only the OPL for intensity measurement did not seem reliable. We also found that the number of GABAa-immunoreactive somas in the INL of rd1 mouse retina (21.5±3.6 cells per frame) was significantly higher than in wild-type (14±1.7; p = 0.002; n = 5; Fig 3C, 3D and 3F).

### GABAc receptor was downregulated following photoreceptor loss in rd1 mouse retina

GABAcR-ρ2 subunit is known to express primarily on bipolar cell axon terminals [34–35]. Western blot analysis revealed that the levels of GABAcR-ρ2 in rd1 mouse retina (0.167±0.04) were significantly lower than in wild-type control (0.334±0.07; p = 0.00008; n = 8; Fig 4), suggesting that GABAcR is downregulated in bipolar cell axon terminals.

## Discussion

Using Western blotting, a sensitive method for protein quantification, we studied the expression levels of several excitatory and inhibitory neurotransmitter receptors in adult rd1 mouse. We found that GluR1, glycine and GABAa receptors were upregulated whereas GABAc receptor was downregulated in rd1 mouse retina. Furthermore, GABAaR was upregulated mainly in the INL where a higher number of bipolar and amacrine cell somas expressed GABAa. To our knowledge, this is the first study reporting changes in the levels of multiple neurotransmitter receptors in a model of retinal degeneration, encompassing major excitatory and inhibitory retinal signaling in adult rd1 mouse, and provides a framework for future studies. For example, it would be interesting to study how the changes observed here in adult rd1 mouse evolve during development, how loss of rods versus cones specifically contributes to these changes, or how the neurotransmitter receptors expressed by specific retinal neurons respond to photoreceptor loss.

Our findings are consistent with earlier reports showing that GluR1 receptor and its phosphorylated product are upregulated in rdta mouse, and that GABA and GAD are upregulated in rd1 mouse [18,33,36]. One of these studies [33], although they did not quantify GABAa, reported stronger labeling for GABAaR in the OPL of rd1 mouse as compared to wild-type, but that the pattern in the IPL was similar to wild-type. Using quantitative immunohistochemistry, we show here that GABAaR levels in OPL and INL combined of rd1 mouse were higher than in wild-type, while the levels in IPL were similar. Our finding that a higher number of cell somas in the INL expressed GABAaR in rd1 mouse is consistent with a previous report that rod bipolar cells dissociated from rd1 mouse show a larger GABAa-mediated response than in the control mouse [37].

One concern was that using β-tubulin as loading control could produce artificially higher levels of the proteins studied here, in the rd1 mouse, because absence of photoreceptors in this mouse would result in reduced levels of β-tubulin. However, β-tubulin and similar proteins, such as β-actin, have been employed previously as loading controls in similar studies [17,19,38]. This is perhaps because the levels of these proteins are not significantly altered following photoreceptor loss, attributable to their limited and non-uniform expression in photoreceptors [39–40]. Furthermore, many cones are still present in adult rd1 mouse [7–8,41], which could contribute to the total retinal β-tubulin. To further address this, we compared the pattern of β-tubulin immunolabeling in wild-type and rd1 mouse retinas, and found that β-tubulin was expressed primarily in the inner retina, most of it in IPL and in nerve fiber layer. Furthermore, the expression of β-tubulin near OPL was more pronounced in the rd1 mouse than in wild-type, possibly indicating the presence of β-tubulin in the remnant photoreceptors (data not shown). In addition, using a different protein (syntaxin-I) and a different mouse model of retinal degeneration (MNU-induced) [6], we compared the effect of using β-tubulin or NF-68 (which does not express in photoreceptors) as loading control on the protein levels following photoreceptor loss. For this, we loaded equal proportions of protein from wild-type and MNU-injected mice [42], and found that although the percent change was higher when we used β-tubulin than when we used NF-68, the difference was not statistically significant (data not shown). That our results from Western blotting matched those from quantitative immunohistochemistry for GABAaR, further confirmed that the results obtained using β-tubulin as loading control were reliable.

The changes in the neurotransmitter receptors reported here must have functional implications for the remnant retinal circuitry in rd1 mouse. In general, our findings of upregulation of GluR1, GlyR and GABAaR are consistent with earlier reports that the synaptic activity in both excitatory and inhibitory networks in the inner retina is enhanced following photoreceptor loss [12–13,16–17]. Increased GABAaR levels in the outer retina could possibly explain the hyperpolarized resting potential in bipolar cells in rd1 mouse [13]. Increased levels of GlyR may facilitate the transfer of oscillatory activity from ON pathway to OFF pathway via the glycinergic AII amacrine cells that has been shown recently [43]. Interestingly, we found that GABAcR, which is expressed primarily in the bipolar cell axon terminals, was downregulated in rd1 mouse. The axon terminals of at least rod bipolar cells have been shown to be disintegrated in rd1 mouse [2]. The reduced levels of GABAcR in bipolar cell axon terminals would lead to their disinhibition, and therefore increased glutamate release. This could explain the hyperactivity that has been reported in RGCs following photoreceptor loss in rd1 mouse [9]. This is also consistent with an earlier report showing increased spontaneous firing in retinal ganglion cells in GABAc-null mouse [44].

While reduced levels of GABAcR can explain higher excitatory activity, it is not clear how it fits with current models to explain the oscillatory activity observed in RGCs following photoreceptor loss. A recent report demonstrated that the intrinsic properties of AII amacrine cells are sufficient to generate oscillatory activity in inner retina of rd1 mouse [45]. However, it is not completely clear how AII amacrine cells start to oscillate in the first place following the loss of photoreceptors. One possibility is that the hyperactivity in bipolar cell axon terminals on one hand and the reduced GABAcR levels on the other, result in a higher activation threshold for GABAergic inhibition. This, combined with the presence of excessive GABA, could result in the GABAergic inhibition of bipolar cell axon terminals becoming phasic, thus leading to repetitive cycles of excitation and inhibition in the bipolar cell-amacrine cell feedback loop [17]. This could possibly initiate the oscillatory activity in the AII amacrine cells which could then be sustained based on their intrinsic properties [45].
